# Sodium‐glucose cotransporter 2 inhibitors in heart failure with chronic kidney disease

**DOI:** 10.1002/ehf2.14095

**Published:** 2022-07-25

**Authors:** Shingo Kato, Nobuyuki Horita, Daisuke Utsunomiya

**Affiliations:** ^1^ Department of Diagnostic Radiology Yokohama City University Graduate School of Medicine Yokohama Japan; ^2^ Department of Cardiology Kanagawa Cardiovascular and Respiratory Center Yokohama Japan; ^3^ Chemotherapy Center Yokohama City University Graduate School of Medicine Yokohama Japan

I read with great interest the study by Pandey *et al*.^1^ They performed a meta‐analysis including four large‐scale randomized controlled trials (RCTs) (DAPA HF, EMPEROR‐Preserved, EMPEROR‐Reduced, and SOLOIST‐WHF) and have shown that sodium‐glucose cotransporter 2 (SGLT2) inhibitors reduce cardiovascular death and heart failure (HF) hospitalization among patients with HF, regardless of left ventricular ejection fraction or diabetes mellitus.^1^ However, we still have a concern not analysed in this study: the efficacy of SGLT2 inhibitor in HF patients with chronic kidney disease (CKD). CKD is one of the most common risk factors of HF, and the presence of CKD is associated with increased morbidity and mortality.^2^ Therefore, HF patients with CKD are at very high risk, but the effectiveness of the SGLT2 inhibitor is not well known for this population. According to these backgrounds, we performed a subgroup meta‐analysis to assess the efficacy of SGLT2 inhibitors for HF patients with or without CKD.

**Figure 1:**
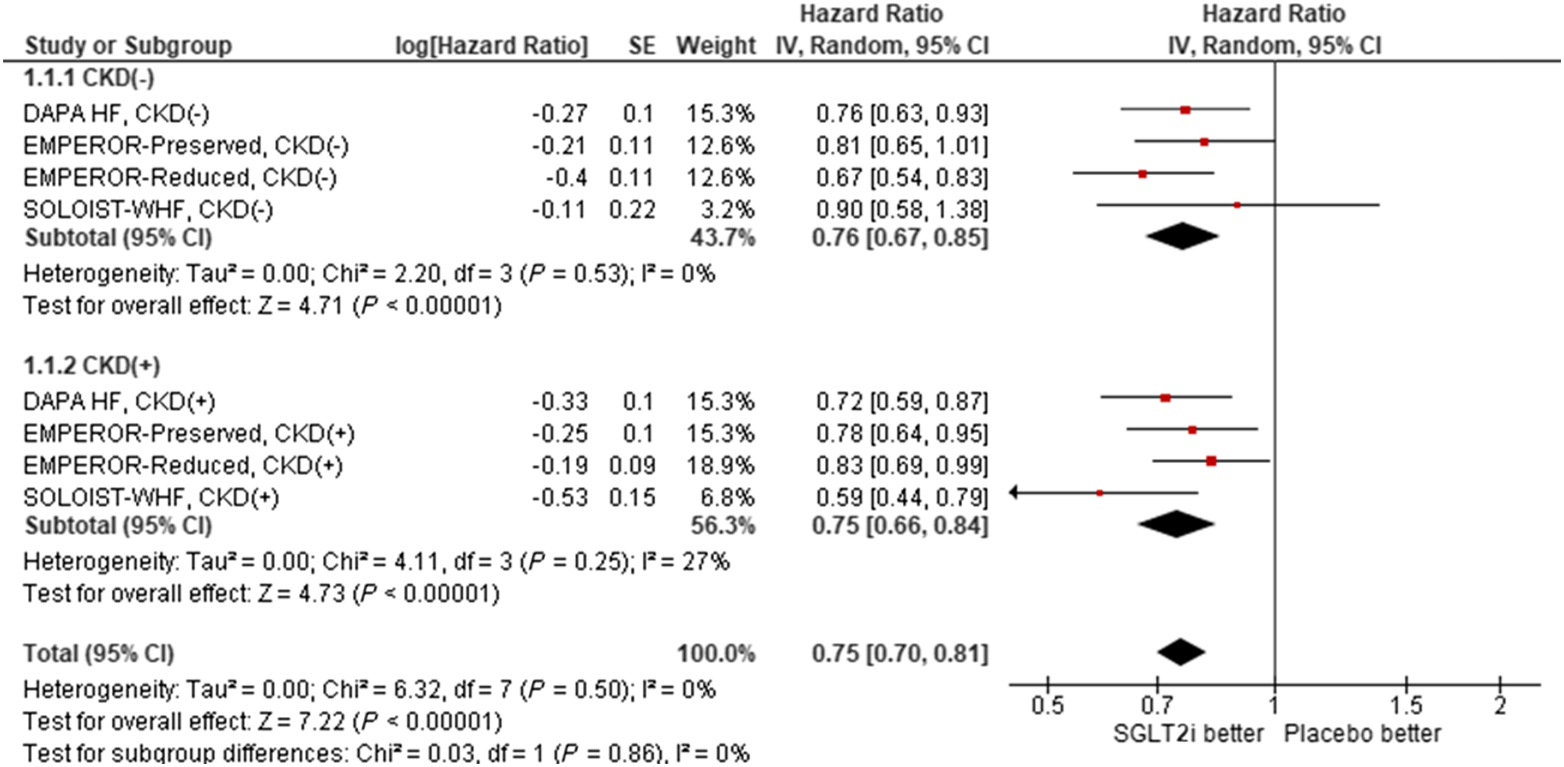
The hazard ratio of composite cardiac events stratified by presence or absence of chronic kidney disease. CI, confidence interval; CKD, chronic kidney disease; SGLT2i, sodium‐glucose cotransporter 2 inhibitors.

We analysed four large‐scale RCTs selected in the paper by Pandey *et al*.^3^, ^4^, ^5^, ^6^ CKD was defined as estimated glomerular filtration rate (eGFR) < 60 mL/min/1.73 m^2^.^7^ A total of 15 678 patients with HF, including 7567 (48%) patients with CKD, were analysed. Two studies used empagliflozin,^3^, ^6^ and each one used dapagliflozin^5^ or sotagliflozin.^4^ Hazard ratio (HR) for primary endpoint (cardiovascular death or hospitalization for HF) was pooled using the random‐model generic inverse variance method after logarithm conversion (RevMan ver 5.4, Cochrane Collaboration, London, UK). *Figure* *1* demonstrates the results of a pooled meta‐analysis. Treatment with SGLT2 inhibitors resulted in a significant reduction in the cardiovascular composite endpoint both in HF patients without CKD [HR: 0.76, 95% confidence interval (CI): 0.67–0.84, *P* < 0.001, *I* ^2^ = 0, *P* for heterogeneity = 0.53] and in HF patients with CKD (HR: 0.75, 95% CI: 0.70–0.81, *P* < 0.001, *I* ^2^ = 27, *P* for heterogeneity = 0.25). The effect of the SGLT2 inhibitor was similar between HF patients with CKD and those without (*P* = 0.86) (*Figure* *1*).

Renal dysfunction frequently coexists with HF, and the presence of both is associated with worse clinical outcomes than either condition alone. Therefore, the patients with coexistence of HF and CKD need aggressive medical treatment. SGLT2 inhibitors are novel glucose‐lowering agents. SGLT2 inhibitors suppress glucose reabsorption by the proximal renal tubules and show an insulin‐independent glucose‐lowering effect.^8^ A recent meta‐analysis demonstrated that the SGLT2 inhibitors significantly reduced adverse cardiac events in patients with HF compared with placebo.^1^ The actual mechanism for the beneficial effect is not completely clear. Several potential theses have been proposed to explain the cardioprotective effects of SGLT2 inhibitor, including diuresis, natriuresis, blood pressure‐lowering, erythropoiesis, and improvement in cardiac energy metabolism.^9^ Our study has shown that the HR of SGLT2 inhibitors is similar between HF patients with CKD and those without CKD. This indicates that the SGLT2 inhibitors effectively prevent adverse cardiac events for HF patients with CKD, who are at very high risk for cardiac events. We believe our data will contribute to considering the indication of SGLT2 inhibitor for HF patients with CKD.
